# The changing landscape of food deserts and swamps in Flanders, Belgium

**DOI:** 10.1093/eurpub/ckac129.758

**Published:** 2022-10-25

**Authors:** V Smets, S Vandevijvere

**Affiliations:** Lifestyle and Chronic Diseases, Sciensano, Anderlecht, Belgium

## Abstract

**Introduction:**

For decades, people's body weight has been increasing at alarming rates, leading to a worldwide obesity epidemic. One of the main causes of this obesity epidemic is poor diet quality. The food environment has been suspected to be one of the principal drivers of poor diet quality. Older people and families with a poor socioeconomic background can be disproportionately affected.

**Methods:**

This study maps the food environment in Flanders between 2008 and 2020 by using the concepts of food deserts and food swamps. Food deserts have been defined as neighborhoods that lack access to some or all foods that are required for a balanced, nutritionally adequate diet. Food swamps refer to places where there is an abundance of unhealthy food options relative to healthy food options. A spatial analysis using population- and retail density datasets yielded the change in food deserts and swamps between 2008 and 2020.

**Results:**

Food deserts in Flanders are found to be small in area and very localized. While food deserts in areas with the two highest deciles of people older than 65 years increased from 1.3% to 1.6% of total surface area in Flanders between 2008 and 2020, the food deserts in areas with the two lowest deciles of low income families decreased from 4% to 2.4%. Food swamps in Flanders on the other hand, are ubiquitous. In 2020, 42.9% of the residential area examined contained no healthy food retailers and 77.7% of the area can be considered a severe food swamp. Areas with a high number of vulnerable groups are healthier than Flanders as a whole because these areas are mostly found in dense urban centers where the ratio of healthy food retailers to all retailers is higher.

**Conclusions:**

Food deserts are a relatively small problem in Flanders in comparison to the widespread occurrence of food swamps. These food swamps exacerbates the obesity epidemic in Flanders and lead to a shorter health span of the affected individuals and to increased medical costs for society.

**Key messages:**

• The food environment in Flanders is generally unhealthy, making it easy for individuals to buy unhealthy foods.

• Food swamps are a major problem in Flanders, where unhealthy retailers drown out healthy retailers.

